# Supplementary material for: Identification of Freezing-Responsive microRNAs and Their Targets in Chinese Jujube by Small RNA and Degradome Sequencing
Source: Int J Mol Sci. 2026 Sep 4;27(17):7912. doi: 10.3390/ijms27177912 (PMC13566890; doi:10.3390/ijms27177912)
Supplement: Supplementary file 1 [file ijms-27-07912-s001.zip › Supplementary Material S2/novel_miR_126.pdf]

Provisional ID : novel_miR_126
Score total : 0.5
Score for star read(s) : -1.3
Score for read counts : 0
Score for mfe : 0.2
Score for randfold : 1.6
Score for cons. seed :
Total read count : 619
Mature read count : 619
Loop read count : 0
Star read count : 0

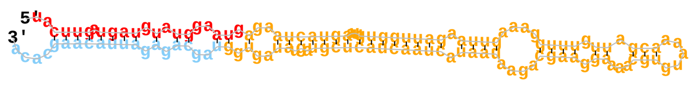

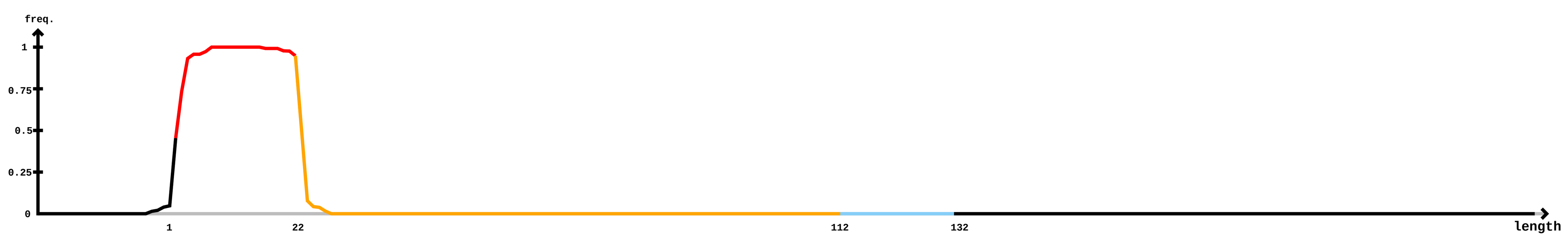

Mature Star

5'- uuggaaaaaaauaucuucuuguacuugaugauguauggaaugagaaucaugaauugugguuagaauugaaagguuuguuagcaaaaugugcaaaaggaagcagaauaauacuaacuacucguaguauagugguagcagagauuacaagcacaaugaaaccauauauuuuggagagauuagauaaagaagagucuuggucucaauuuaaaaauguggcauuuaggcaugaucaaaugucuaaugauuccaa -3' exp

((((((........(((((((.((((.((((.(.((..((.(..(((((((...((((((((.((((....((((.((.(((.....)))...)).))))....)))).)))))))))))).).))..).))..)).).)))))))).)))).))).((((((.((((((((((((((((.......))).)))))))..)))))))))))))((((.((((((......))))))))))).))))))

	reads	mm	sample
.................cuuguacuugaugauguau	2	0	JCK-2
.................cuuguacuugaugauguau	1	0	JCK-3
.................cuuguacuugaugauguau	2	0	JCK-1
.................cuuguacuugaugauguauggaau	4	0	LCK-3
..................uuguacuugaugauguauggaau	3	0	JCK-3
..................uuguacuugaugauguauggaaug	1	0	JCK-1
...................uguacuugaugauguauggaaug	5	0	JCK-2
...................uguacuugaugauguauggaaug	2	0	JCK-3
...................uguacuugaugauguauggaaug	2	0	JCK-1
...................uguacuugaugauguauggaauga	3	0	LT-3
....................guacuugaugauguauggaaug	4	0	JCK-2
....................Cuacuugaugauguauggaaug	1	1	JCK-1
.....................uCcuugaugauguaugga	1	1	LT-2
.....................uacuugaugauguaugga	4	0	LT-3
.....................uacuugaugauguaugga	4	0	LT-2
.....................uacuugaugauguauggaa	1	0	LCK-3
.....................uacuugaugauguauggaau	4	0	LT-1
.....................uacuugaugauguauggaau	1	0	JCK-3
.....................uacuugaugauguauggaUu	1	1	LT-3
.....................uacuugaugauguauggaau	3	0	LT-3
.....................uacuugaugauguauggaaug	44	0	JCK-2
.....................uacuugaugauguauggaaug	35	0	JCK-3
.....................uacuugaugauguauggaaug	24	0	LT-3
.....................uacuugaugauguauggaauU	3	1	LT-2
.....................uacuugaugauguauggaauU	2	1	LT-1
.....................uacuugCugauguauggaaug	1	1	LT-3
.....................uacuugaugauguauggaaug	11	0	LCK-3
.....................uacuugaugauguauggaauU	10	1	LCK-3
.....................uacuugaugauguauggaauU	1	1	JCK-2
.....................uacuugCugauguauggaaug	1	1	JCK-1
.....................uacuugaugCuguauggaaug	1	1	JCK-2
.....................uacuugaugauguauggaaug	17	0	JCK-1
.....................uacuugaugauguauggaaug	34	0	LT-1
.....................uacuugaugauguauggaauU	5	1	LT-3

Mature   Star

```
uuggaaaaaaauaucuucuuguacuugaugauguauggaaugagaaucaugaauugugguuagaauugaaagguuuguuagcaaaaugugcaaaaggaagcagaauaauacuaacuacucguaguauagugguagcagagauuacaagcacaaugaaaccauauauuuuggagagauuagauaaagaagagucuuggucucaauuuaaaaauguggcauuuaggcaugaucaaaugucuaaugauuccaa
....................uacuugaugauguauggaaug.......     26   0   LT-2
....................uacuugaugauguauggaauga......     4    0   JCK-3
....................uacuugaugauguauggaauga......     1    0   LT-2
....................uacuugaugauguauggaauga......     7    0   JCK-2
....................uacuugaugauguauggaauga......     7    0   JCK-1
....................uacuugaugauguauggaaugU......     1    1   JCK-1
....................uacuugaugauguauggaauga......     2    0   LT-1
....................uacuugaugauguauggaaugaU.....     1    1   JCK-2
.....................acuugaugauguauggaaug.......     8    0   JCK-3
.....................acuugaugauguauggaaug.......     9    0   LT-1
.....................acuugaugauguauggaaug.......     2    0   JT-2
.....................acuugaugauguauggaaug.......     4    0   LT-3
.....................acuugaugauguauggaaug.......     6    0   JT-3
.....................acuugaugauguauggaaug.......     8    0   JCK-2
.....................acuugaugauguauggaaug.......     8    0   JCK-1
.....................acuugaugauguauggaaug.......     7    0   LCK-3
.....................acuugaugauguauggaaug.......     2    0   LT-2
.....................acuugaugauguauggaauga......     22   0   JCK-3
.....................acuugaugauguauggaauga......     42   0   JCK-2
.....................acuugaugauguauggaauga......     7    0   LT-3
.....................acuugaugauguauggaauga......     10   0   LCK-3
.....................acuugaugauguauggaauga......     7    0   LT-2
.....................acuugaugauguauggaaugU......     1    1   LT-1
.....................acuugaugauguauggaauga......     26   0   JCK-1
.....................acuugaugauguauggaauga......     8    0   LT-1
......................cuugaugauguauggaau........     1    0   LT-3
......................cuugaugauguauggaau........     1    0   LT-2
......................cuugaugauguauggaaug.......     1    0   JCK-2
......................cuugaugauguauggaaug.......     2    0   JCK-1
......................cuugaugauguauggaaug.......     3    0   LT-3
......................cuugaugauguGuggaaug.......     2    1   JCK-2
......................cuugaugauguauggaaug.......     4    0   LT-1
......................cuugaugauguGuggaaug.......     1    1   JCK-1
......................cuugaugauguauggaaug.......     5    0   LT-2
......................cuugaugauguauggaauU.......     1    1   LT-2
......................cuugaugauguauggaaugU......     1    1   LT-1
......................cuugaugauguauggaauga......     11   0   JT-1
......................cuugaugauguauggaauga......     10   0   LT-1
......................cuAgaugauguauggaauga......     1    1   LT-2
......................cuugaugauguauggaauga......     9    0   LT-3
......................cuugaugauguauggaauga......     16   0   LT-2
......................cuugaugauguauggaauga......     4    0   JCK-3
......................cuugaugauguauggaauga......     4    0   JCK-1
......................cuugaugauguauggaauga......     21   0   LCK-3
......................cuugaugauguauggaauga......     9    0   JCK-2
......................cuugaugauguauggaaugaU.....     2    1   JCK-1
......................cuugaugauguauggaaugaC.....     1    1   JCK-2
......................cuugaugauguauggaaugaC.....     2    1   LT-3
......................cuugaugauguGuggaaugag.....     1    1   LT-3
......................cuugaugauguauggaaugaC.....     3    1   LT-1
......................cuugaugauguauggaaugaU.....     1    1   JCK-2
......................cuugaugauguauggaaugaU.....     5    1   LT-2
......................cuugaugauguauggaaugaU.....     1    1   JCK-3
......................cuugaugauguauggaaugaU.....     2    1   LT-3
.......................uugaugauguauggaaug.......     2    0   LT-2
.......................uugaugauguauggaaug.......     1    0   LCK-3
.......................uugaugauguauggaaug.......     2    0   JT-2
.......................uugaugauguauggaaug.......     4    0   JT-3
.......................uugaugauguauggaauga......     1    0   LT-3
.......................uugaugauguauggaauga......     1    0   JCK-3
.......................uugaugauguauggaauga......     1    0   LT-1
.......................uugaugauguauggaauga......     1    0   JCK-2
.......................uugaugauguauggaaugaU.....     1    1   JCK-2
.......................uugaugauguauggaaugaU.....     1    1   JCK-1
.......................uugaugauguauggaaugaU.....     1    1   LT-1
.........................gaugauguauggaaugagaau..     3    0   JT-3
.........................gaugauguauggaaugagaau..     7    0   JT-2
..........................augauguauggaaugaga....     1    0   JCK-3
..........................augauguauggaaugaga....     1    0   LT-2
..........................augauguauggaaugaga....     1    0   JCK-1
```

```
                    Mature                                                                                              Star
uuggaaaaaaauaucuucuuguacuugaugauguauggaaugagaaucaugaauugugguuagaauugaaagguuuguuagcaaaaugugcaaaaggaagcagaauaauacuaacuacucguaguauaguggaugaaaccauauauuuuggagagauuagauaaagaagagucuuggucucaauuuaaaaauguggcauuuaggcaugaucaaaugucuaaugauuccaa
...........................augauguauggaaugagaa............................................................................................................................................................................................ 1 0 JCK-2
...........................augauguauggaaugagaa............................................................................................................................................................................................ 1 0 LT-3
...........................augauguauggaaugagaC............................................................................................................................................................................................ 1 1 JT-2
...........................augauguauggaaugagaa............................................................................................................................................................................................ 2 0 JCK-1
...........................augauguauggaaugagaa............................................................................................................................................................................................ 7 0 JT-2
...........................augauguauggaaugagaa............................................................................................................................................................................................ 2 0 JT-3
```
